# Enhanced molecular recognition with longer chain crosslinkers in molecularly imprinted polymers for an efficient separation of TR active substances†

**DOI:** 10.1039/d3ra08854e

**Published:** 2024-04-15

**Authors:** Takuya Kubo, Mayuko Yagishita, Tetsuya Tanigawa, Sayaka Konishi-Yamada, Daisuke Nakajima

**Affiliations:** a Division of Applied Life Sciences, Graduate School of Life and Environmental Sciences, Kyoto Prefectural University 1-5 Shimogamo Hangi-cho, Sakyo-ku Kyoto 606-8522 Japan tkubo@kpu.ac.jp; b Department of Material Chemistry, Graduate School of Engineering, Kyoto University Katsura, Nishikyo-ku Kyoto 615-8510 Japan; c Department of Life and Environmental Science, Prefectural University of Hiroshima Shobara City Hiroshima 727-0023 Japan; d Health and Environmental Risk Division, National Institute for Environmental Studies (NIES) Tsukuba City Ibaraki 305-8506 Japan

## Abstract

Molecular imprinting technology has been widely studied as a technique to obtain molecular recognition by artificial means. Selecting functional monomers or polymerization conditions plays a key role to optimize molecularly imprinted polymer (MIP) synthesis. To date, there have been few reports well exploiting the effect of crosslinkers. Here, in this study, we synthesized the MIPs using poly(ethyleneglycol) dimethacrylate with different units of ethylene oxide (*n* = 1 to 23) as crosslinkers to observe the molecular recognition abilities. The MIPs were attached to the surface of mono-dispersed polymer beads. The obtained spherical MIPs and non-imprinted polymers were filled in a column for high performance liquid chromatography. Then the retention selectivity toward TR active substances was evaluated. The result revealed that the recognition ability did not improve regardless of the amount of ethylene oxide. With the crosslinker (*n* = 9), extremely high retention selectivity was observed, which provides at most around ten times as large imprinting factors in comparison with other MIPs. Interestingly, we obtained the highest recognition ability at around polymerization temperature from the evaluation of the recognition ability toward temperature shift using the MIP (*n* = 9). In general, hydrogen bonding based on MIPs provides high recognition ability at low temperature, whereas, this study indicates that the use of flexible crosslinkers may enable the synthesis of MIPs that precisely memorize the conditions of polymerization. Lastly, we simultaneously analyzed the TR active substances using the column filled with MIPs (*n* = 9). The result showed relatively linear correlation between the retention strength of each substance and phycological activity toward TR obtained by yeast assay. Therefore, we can conclude that an induced fit like the receptor emerged by constructing the flexible molecular recognition field.

## Abstract 2

We report the effects of the crosslinker length on the molecular recognition for the preparation of molecularly imprinted polymers.

## Graphical abstract

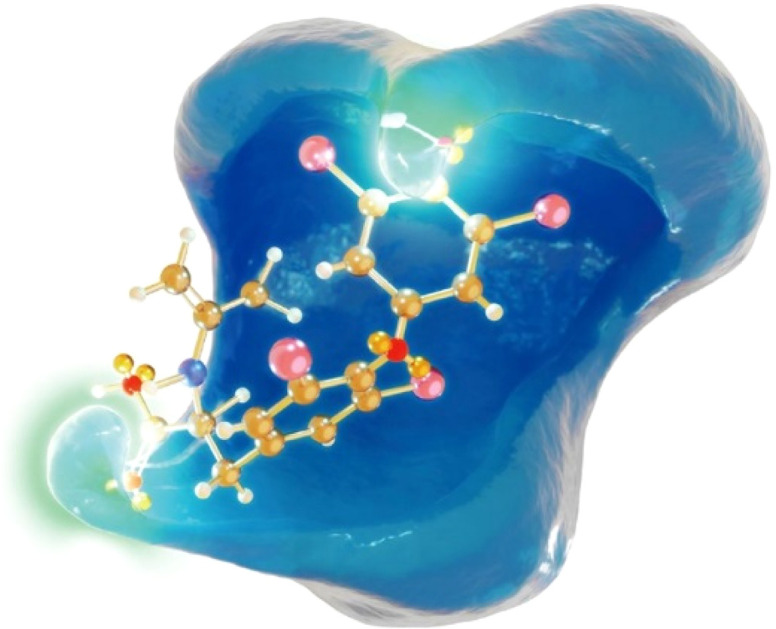

## Introduction

Molecular imprinting technology is one of the methods for obtaining artificial molecular recognition materials.^1–6^ It polymerizes a target molecule as a template molecule under the coexistence of functional monomers and a crosslinker, which enables the construction of a key and a key hole at the molecular level. This technology expands in a broad range of fields, such as separation media for chromatography,^7–9^ sensors^10–15^ and artificial catalysts^16–20^ due to its easier operation for obtaining selective molecular recognition. At the beginning, most studies targeted low-molecular weight compounds. Nowadays, molecularly imprinted hydrogels have been employed toward large molecules such as proteins.^21–24^

Generally, selecting optimum functional monomers toward target molecules is of high importance in molecular imprinting technology. To make full use of the interaction between target molecules and functional monomers, the optimal ones should be considered from a variety of solvents; the most optimum functional monomers are selected using spectrum analysis such as NMR. Molecularly imprinted polymers (MIPs), which were obtained through such processes above, show selective molecular recognition toward target molecules and structurally related substances. In the case of relatively rigid polymer matrixes, molecular recognition sites are rigor, which are commonly employed. However, ideal recognition sites should be flexible such as receptors *in vivo*. Most MIPs previously reported have lower molecular recognition ability toward receptors, which is far from obtaining ideal artificial molecular recognition. Here, we focus on the spacer length of crosslinkers to improve molecular recognition on MIPs. When target molecules approach, the receptors consisting of proteins normally acquire target molecules adjusting their forms, which means that induced fit occurs. Therefore, if the same function was replicated using artificial materials, high selective molecular recognition comparable to receptors would realize. In the previous study, we have accomplished an efficient screening method for estrogen receptor (ER) active substances using the MIP-based media imitating receptors and the accurate mass spectrometry measurement.^25^ In development of bioactive specific media, we have solved low qualitative and quantitative capability of conventional media caused by non-specific adsorption due to hydrophobicity. Consequently, we achieved to develop a novel medium that is beneficial for selective separation and condensation of ER active substances in actual environment analytes. The study of ER may be effective for various endocrine disruption chemicals (EDCs)^26–28^ involving influential mechanisms *via* receptors and can be the first screening method for chemical substances and environmental analytes. Regarding the current environmental policy of EDCs, Ministry of the Environment (MOE) announced EXTEND 2022 as “a plan for acting of endocrine disruption of chemical substances” in 2022.^29^ This program made a significant change on evaluation for the acting of endocrine disruption as a thyroid hormone (TH) in comparison with EXTEND 2010 and 2016. This change can be related with another program (JECS) that Ministry of the Environment has been working. JECS revealed that TH abnormality affects the brain and increases childhood developmental disorder as well as a woman with TH abnormality can be at risk of having a malformed fetus. Moreover, Environmental Protection Agency (EPA) and Endocrine Society also make a statement of effects on ECDs to a number of disorders, such as cancer of thyroid, thyroid disease, obesity and hypertension. *Via* an academic perspective, polychlorinated biphenyls (PCBs), their derivatives and brominated aromatic series show activated thyroid receptor (TR). It concerns that countless candidate substances can exist in chemical products and environment. Under these backgrounds, “evaluation of effects on thyroid” indicated in EXTEND 2022 suggests specification of candidate substances based on an academic evaluation, experiments *in vitro*, and experiments *in vivo*. Some candidate substances are identified in estrogens and androgens but not in TR active substance. Namely, TR active substance does not offer enough substances for a biological experiment. EPA, European Environment Agency and MOE have lately recommended “New Approach Methodologies” instead of existing biological methods. A novel screening method to effectively identify TR active substances using engineering approaches is thus desirable in any case from upper stream to down-stream, *i.e.* chemical products and novel chemical substances, biological (exposure), egestion, and environmental samples (drainage).^30–36^

In this study, we indicate the possibilities to obtain molecular recognition similar to receptors and realize an effective screening method for TR active substances by solving the problems of a MIP previously described. MIPs were synthesized using poly(ethylene glycol) dimethacrylate with different units of ethylene oxide (*n* = 1 to 23) as crosslinkers. Then the retention selectivity was evaluated toward the template molecule by filling the obtained MIPs in the column for high performance liquid chromatography (HPLC). Next, an adsorption experiment at different temperatures was conducted to evaluate effects of polymerization state when synthesizing MIPs. Lastly, we simultaneously analyzed TR active substances using the MIPs that showed the highest selective molecular recognition. By evaluating the correlations between retention strength on HPLC and TR physiological activity *via* the yeast assay previously reported,^37,38^ we examined a possibility of a screening for TR active substances.

## Methods

### Fabrication of surface-modified MIP particles

The use of packed agents involving recognition sites on the surface of a particle is preferable to maximize molecular recognition ability of the MIP. Meanwhile, with a multistep swelling and polymerization method, one of the methods for synthesizing monodispersed spherical particles, lots of unreacted residual vinyl groups exist when polymer particles are synthesized using a crosslinker alone. In this study, we synthesized so-called core–shell type MIP particles, which construct MIP layers on the surface of polymer particles synthesized with only EDMA as the core.

First, as previously reported, particles were synthesized using toluene as a solvent to make pores from polystyrene seeds and EDMA as a crosslinker.^39^ Then PEG based crosslinkers (poly(ethylene glycol) dimethacrylate, PEDMA, ethylene oxide unit = 1, 4, 9, 14, 23, Fig. S1†) corresponding to 10 wt% toward 0.5 g of EDMA seeds, AcetylT4 (AcT4, 28 μmol) as a template molecule and 4-vinylpyridine (4Vp) (112 μmol) as a functional monomer were mixed in a polymerization solvent, a mixture of acetonitrile/DMSO = 2/1 (v/v, 1.5 mL). After argon bubbling, the mixture was polymerized for 18 h agitating ADVN (40 mg) as an initiator at polymerization temperature, 60 °C. In the previous study, the MIP using AcT4 as a template molecule and 4Vp as a functional monomer showed high adsorption selectivity toward TR biding substances.^40^ After finishing polymerization, the solvents were removed by centrifugal separation. And then the particles were dispersed in the washing solvents, including 5% formic acid aq. contained ethanol, pyridine, acetone of each 10 mL with ultrasonication for 30 min to remove template molecules and any unreacted regents. Using the same method, non-imprinted polymer (NIP) particles not having a template molecule were synthesized. At the same time, MIP using TRIAC as a template molecule and NIP particles were synthesized for comparison. The compositions of all the polymers are summarized in Table S1.†

### Fabrication of packed columns with MIP particles and HPLC measurement

After washing and drying, MIP particles of 0.5 g ultrasonically were dispersed in toluene/2-propanol = 1/1 (v/v) of 10 mL as a slurry solvent were filled in an HPLC column (an inner diameter of 2 mm and a length of 50 mm) by pressurizing using methanol with 35 bar. Then the columns were washed with 0.1% formic acid aq./methanol = 2/8 as a mobile phase to remove the template molecules completely using HPLC with UV detection at 230 nm which is the specific wavelength of AcT4. After the base-line became a plateau, the columns were evaluated in HPLC using several mobile phases at different temperatures to evaluated the separation selectivity. The concentrations of analytes were 100 mg mL^−1^ in each mobile phase, and the temperature was controlled by the column oven of HPLC instrument. The detailed HPLC conditions are described in each figure caption. Finally, several retention behaviors of TR active substances were also evaluated using the column that showed the highest retention selectivity toward AcT4.

### Adsorption isotherm

To evaluate the binding strength of the prepared MIP toward AcT4, a batch adsorption test was carried out. Standard AcT4 solutions in acetonitrile were prepared with concentration at 0.1 to 1500 μM. The 9G-MIP of 10 mg was dispersed in AcT4 solutions of 1.0 mL and stirred at 40 °C for 12 h. After centrifugation, the supernatants were evaluated by HPLC to determine free AcT4 and the adsorbed AcT4 was estimated. The HPLC conditions were employed as following: column, Quichsorb 5 μm (150 × 2.1 mm i.d.) (ChemcoPlus, Osaka, Japan); mobile phase, (A) 0.1% formic acid aq., (B) 0.1% formic acid in acetonitrile, gradient 15% B (0–2 min), 70% B (2–7 min), 15% B (7–9 min); flow rate, 0.2 mL min^−1^; temperature, 40 °C; detection, UV 230 nm (high conc.) and MS-SIM negative mode *m*/*z* 817.91 (low conc.).

## Results and discussion

### MIP coated polymer particles

A MIP is generally utilized through following steps: synthesizing a bulk polymer, crushing, and classifying. However, even after MIPs are crushed and classified, their shapes are still random, and size distribution is still wide. It makes separation ability extremely low and the pressure when solution is delivered relatively unstable and high. In our previous study, the core–shell type MIPs for TR active compounds were prepared. Briefly, the MIP with AcT4 as a templet molecule, 4Vp as a functional monomer and EDMA as a crosslinker were modified to monodispersed silica spherical particles. During polymerization, the template competitively interacts with not only functional monomers but also a silanol group in the MIP modified particles on the surface of a silica particle. This interaction may heterogeneously form recognition site, and thus, the column did not show sufficient imprinting effects.^40^ Here, we fabricated MIP coated polymer particles that non-specific interaction does not emerge as MIP recognition sites are formed on the surface of EDMA polymer particles, the particle size of 5 μm prepared by multistep swelling and polymerization. These particles have narrow size distribution, which makes pressure resistance and separation efficiency high. Moreover, they are not affected by the secondary interaction caused by such as a silanol, expected to be an ideal medium involving homogenic recognition sites and high separation efficiency.

**Fig. 1:**
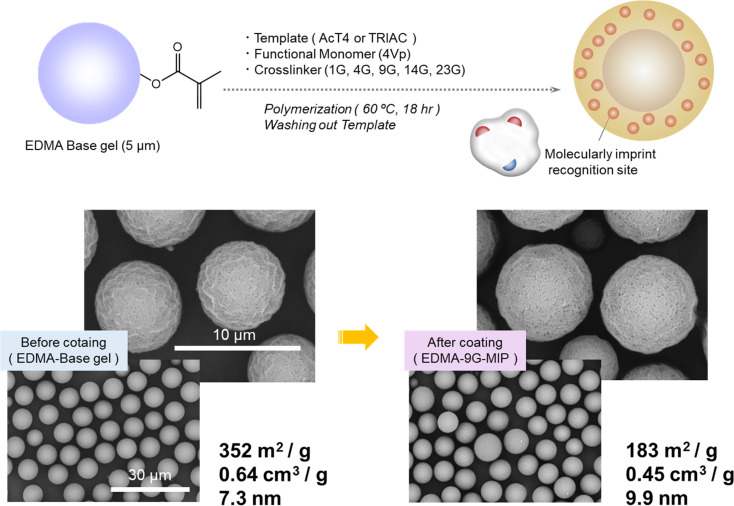
Preparation of core–shell type MIP particles.

Then we performed MIP coating to the core made of EDMA seeds. Fig. 1 shows the results of SEM images and nitrogen adsorption measurement using 9G as a crosslinker of MIP coating. These SEM images represent that the surface of the particles became smooth, and the whole particle itself got a bit bigger after coating. According to the results of gas adsorption, specific surface area and pore volume both decreased after modification with the MIP layer. As the morphological changes were observed, it can conclude that surface modification was successfully achieved.

### MIP coated particles using crosslinkers having different chain lengths

**Fig. 2:**
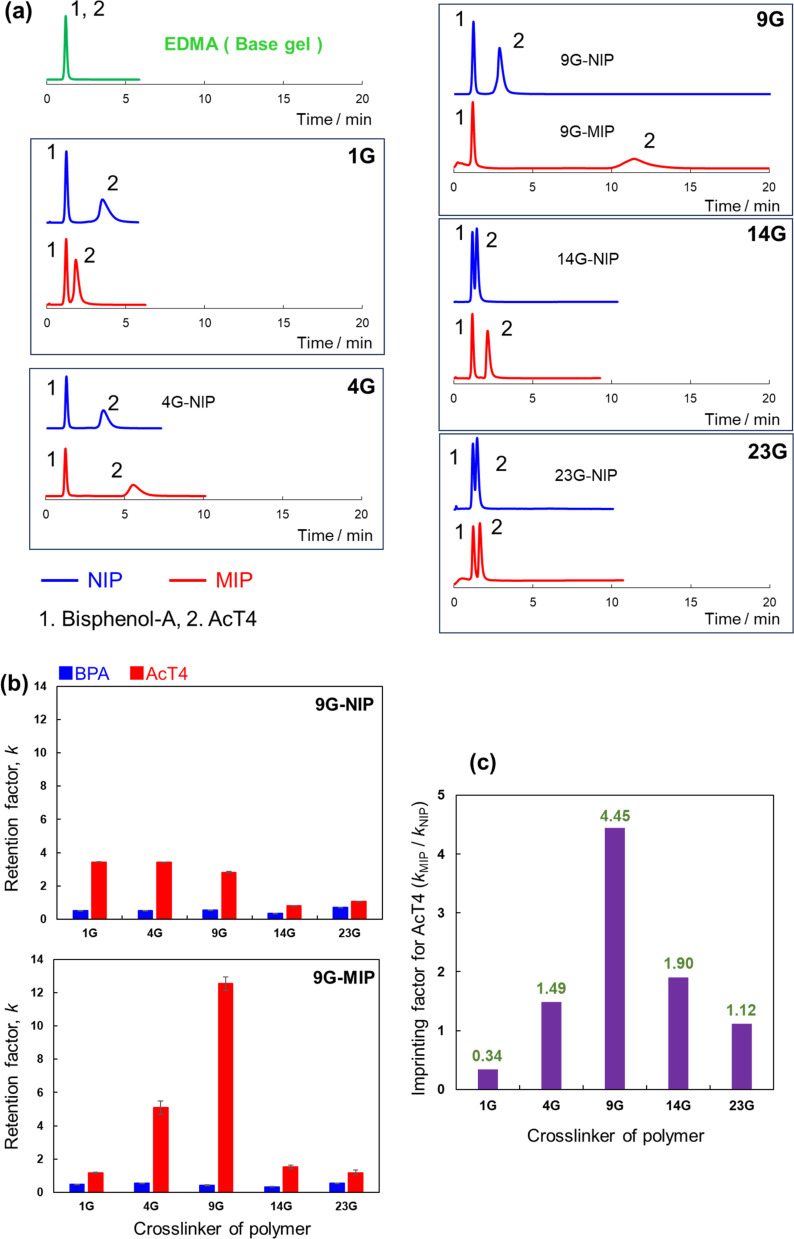
Retention of BPA and AcT4 with the prepared MIPs and NIPs. (a) Chromatograms for each column, (b) retention factors, (c) imprinting factors. HPLC conditions: analytes, BPA + AcT4 (0.1 mg mL^−1^, 5 μL); column size, 2.0 mm i.d. × 50 mm; flow rate, 0.2 mL min^−1^; detection, UV 240 nm; temperature, 40 °C; mobile phase, 0.2% HCOOH in 90% MeOH/acetonitrile = 10/90 (v/v).

Table 1 showed the morphological differences of MIPs. The specific surface areas, pore volumes, and average pore size were strongly affected by the chain length of crosslinkers. Therefore, we anticipated that various chain lengths of crosslinkers should contribute to the molecular recognition abilities. We evaluated retention selectivity toward a template molecule by packing the MIP particles and NIP particles into an HPLC column. The chromatogram with the mixed analytes of BPA and AcT4 is shown in Fig. 2(a). Consequently, retention of AclT4 with both MIP and NIP was increased by 4Vp as a functional monomer, although absolute retention was small with 1G-MIP, and imprinting effect was not observed.

**Table 1:** Morphological parameters by N_2_ adsorption tests

Polymer	Specific surface area (m^2^ g^−1^)	Total pore volume (cm^3^ g^−1^)	Average pore size (nm)
EDMA (base gel)	352	0.64	7.3
1G-MIP	322	0.63	7.8
4G-MIP	247	0.50	8.0
9G-MIP	183	0.45	9.9
14G-MIP	177	0.51	11.5

Imprinting effect on MIP was not observed with 1G as described above, which may be related to the length of a crosslinker. 1G (same as EDMA) is normally a suitable crosslinker for MIP toward compounds with the relatively low-molecular weight. We anticipate that a crosslinker with a short chain length cannot obtain enough space to construct recognition sites because the template molecule that we applied in this study involves comparatively bigger molecular size and a flexible structure.

Then we fabricated the similar MIP coated particles using PEDMA having different ethylene oxide unit number, (*n* = 1, 4, 9, 14, 23), evaluating them by HPLC. These results are also shown in Fig. 2(a). The obtained chromatograms confirmed imprinting effect on AcT4 with all the MIPs except for the 1G-MIP. Very curiously, selectivity toward AcT4 gets higher up to *n* = 9. In contrast, it gradually declines above *n* = 9. These results suggest that more flexible recognition sites effectively take in AcT4 at *n* = 9 and provides superior recognition sites.

To summarize the difference of the molecular recognitions, the retention factor, *k*, and imprinting factor (IF) are compared in Fig. 2(b and c). The retention factor and imprinting factor are defined as follows:*k* = (*R*_*t*_0__ − *R*_anal_)/*R*_*t*_0__, IF = *k*_MIP_/*k*_NIP_where, *R*_*t*_0__, *R*_anal_, *k*_MIP_, and *k*_NIP_ are retention time of dead volume, retention time of the analyte, retention factor on MIP, and retention factor on NIP, respectively. According to these results, the imprinting effect is clearly corresponding to the length of the crosslinker. The 9G-MIP consisting of the relative longer chain crosslinker showed the greatest selectivity, while the use of exclusively longer chain length makes recognition site loose, which means that precise molecular recognition is not achieved. We anticipated that the polymer matrix constructed with a suitable length crosslinker provides the flexible molecular recognition sites such like proteins. As further optimization, the degree of modification of the MIP to core EDMA particles should be evaluated. Then additional 9G-MIPs were prepared by decreasing and/or increasing the amount of the crosslinker as shown in Table S1.† The polymer particles were also packed into the column and evaluated by HPLC. The results are shown in Fig. S2.† As we expected, the recognition abilities for AcT4 in both 9G-MIP-less and 9G-MIP-more were significantly lower than that of the 9G-MIP. We supposed that the use of the low amount of crosslinkers did not provide enough recognition sites, and the excess amount of crosslinkers filled the recognition sites with higher density of the polymer matrix. Consequently, the suitable molecular recognition ability was obtained with the 9G-MIP prepared with the moderate amount of the crosslinker.

To date, there have been no reports that a chain length varies molecular recognition. It should be thus developed as a novel optimized factor for MIP synthesis. This phenomenon mimics induced-fit molecular recognition primarily demanded in MIP study, and it may play a principal role in further MIP study.

To ensure our perception, we evaluated similar MIP coated particles using another template molecule, TRIAC. TRIAC is the compound that we have reported recently as a TR active substance found from the environment. It is also very interesting compound as the substance that cannot pass through the brain barrier in animal experiments.^41^ As TRIAC is slightly smaller than AcT4, we anticipated that TRIAC shows different recognition ability from AcT4. Based on the composition in Table S1,† we also evaluated 9G-MIP-TRIAC particles by HPLC. The result is shown in Fig. S3.† As we expected, the retention selectivity toward TRIAC increased as the chain length became longer, indicating that an optimized length of a crosslinker must exist. Meanwhile, when we examined retention of TRIAC with 9G-MIP described above, imprinting effect also emerged due to its smaller retention than 9G-MIP-TRIAC.

### Molecular recognition at different temperatures

**Fig. 3:**
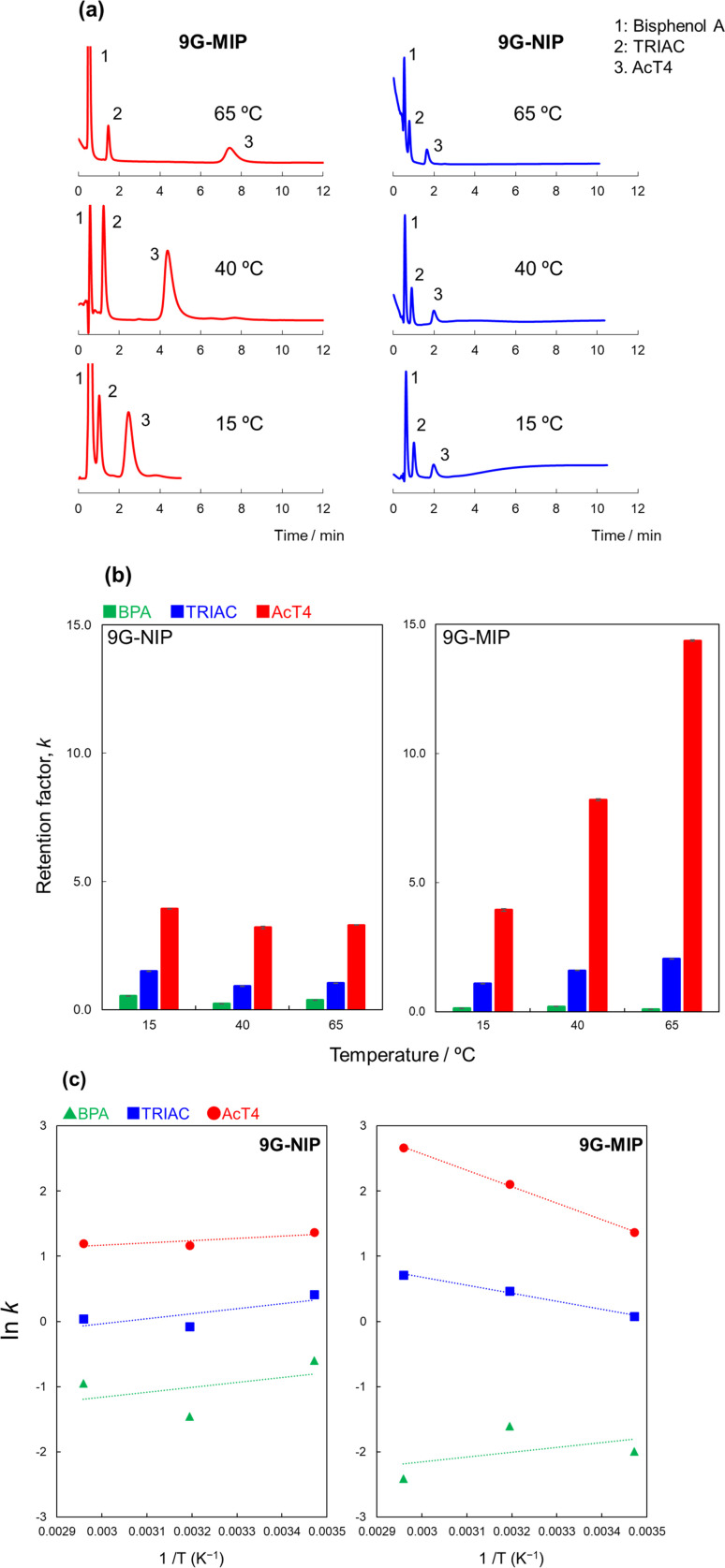
Retention of BPA, TRIAC, and AcT4 under different temperature. (a) Chromatograms for each column, (b) retention factors, (c) the van't Hoff's plot. HPLC conditions: analytes, BPA + AcT4 (0.1 mg mL^−1^, 5 μL); column size, 2.0 mm i.d. × 50 mm; flow rate, 0.2 mL min^−1^; detection, UV 240 nm; temperature, 40 °C; mobile phase, 0.2% HCOOH in 90% MeOH/acetonitrile = 10/90 (v/v).

As shown above, the MIP prepared with the moderated crosslinker showed significant higher selectivity to the template molecule. To understand the molecular recognition ability of the MIP, the effect of temperature was evaluated. In typical HPLC, at higher temperature, the retention decreases due to lower viscosity of mobile phases, and weaken intermolecular interaction such as hydrogen bonding When the van't Hoff's equation^42–44^ is applied to HPLC results, a simple consideration is described as belowln *k* = −(1/*RT*)Δ*H*° + (1/*R*)Δ*S*° + ln *Φ*where *k*, *R*, *T*, Δ*H*°, Δ*S*° and *Φ* are retention factor, gas constant [J mol^−1^ K^−1^], absolute temperature [K], standard enthalpy [kJ mol^−1^], standard entropy [J mol^−1^ K^−1^], and the phase ratio (the ratio of stationary and mobile phase volumes within the column). The 9G-MIP and the 9G-NIP were evaluated by HPLC at different temperatures. Each chromatogram and the retention factors are summarized in Fig. 3(a and b). As we expected, the retentions of all analytes decreased at lower temperature in the 9G-NIP. In contrast, the retention of AcT4 clearly increased at higher temperature in the 9G-MIP. These results are considered with the van't Hoff's equation as shown in Fig. 3(c). Making phase ratios, *Φ*, and constant simplifies the equation in the both MIP and NIP. In the 9G-NIP, the linear relation with ever-increasing was obtained, indicating the retention mechanism is dominated by the enthalpy as well as typical HPLC. On the other hand, TRIAC and AcT4 were considered as a backward linearity in the 9G-MIP, and bisphenol A showed the relation similar to that of the 9G-NIP. The results of TRIAC and AcT4 on the 9G-MIP did not seem enthalpy domination but entropy domination. In authentic MIPs, the molecular recognition sites are significantly rigid so that the recognition ability should stay the same at even different temperatures, and the retention strength should depend on enthalpy. In the case of our study, the polymer matrix was relatively flexible and the recognitions sites were also movable. When the MIP was prepared at 60 °C, the recognition sites should be the most stable at around the polymerization temperature. According to these results, we were able to construct the flexible recognition sites in the MIP, and the MIP memorizing the 3D shape of the template molecule at around the polymerization temperature, such like an induced-fit type molecular recognition.

### Adsorption isotherm

**Fig. 4:**
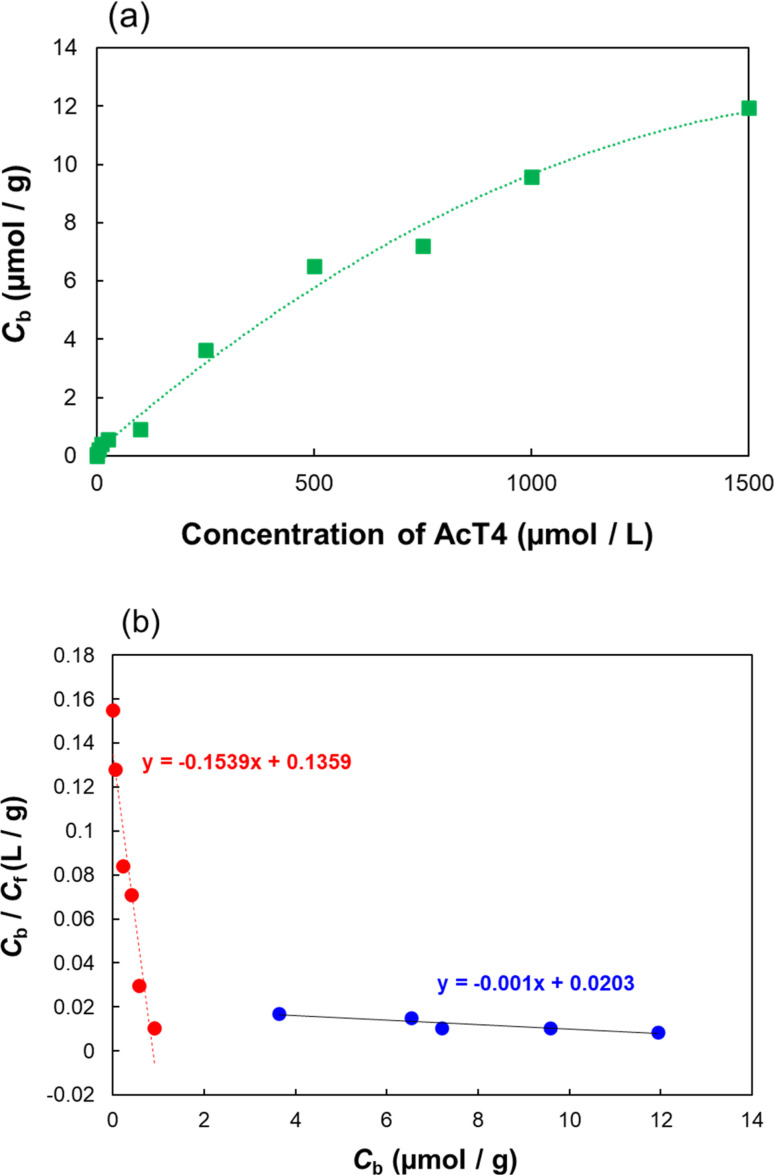
Adsorption isotherm (a) and Scatchard plots (b) on the 9G-MIP.

Additionally, in order to know the quantitative binding strength toward AcT4 with the 9G-MIP, the isotherm adsorptions were evaluated. To evaluate the binding properties, we employed Langmuir model to estimate a few parameters.^45–47^ Langmuir model showed the typical results due to the imprinting sites, which usually show bimodal recognition sites containing high and low affinity. The amount of adsorption and Scatchard plots of batch adsorption are shown in Fig. 4. The adsorption behavior over the entire AcT4 concentrations could be explained using two binding constants. Similar behavior is observed in MIP studies,^48–52^ in brief, the higher binding constant of 1.6 × 10^5^ M^−1^ and lower binding constant of 1.0 × 10^3^ M^−1^. The results clearly confirmed the existence of selective molecular recognition sites in the 9G-MIP.

The Scatchard plots are prepared by Langmuir model and the following equation,*C*_b_/*C*_f_ = *nK*_P_ − *KC*_b_,where *C*_b_, *C*_f_, *n*, and *K* are the amount of bounded MTX, the free concentration of AcT4 in solution after the equilibrium, the number of the binding sites, and the binding constant with AcT4, respectively.

### Molecular recognition to TR active compounds

**Fig. 5:**
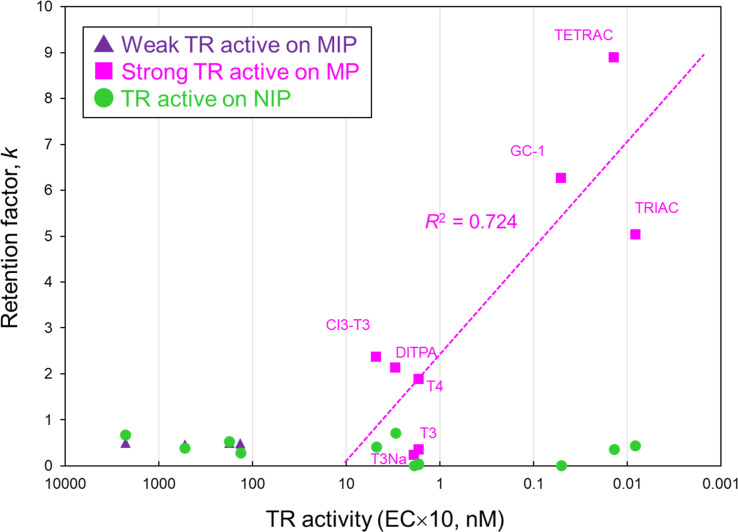
Correlation between retention strength on MIP/NIP and phycological activity toward TR. HPLC conditions: analytes, 0.1 mg mL^−1^ each analyte, 4 μL; column size, 2.0 mm i.d. × 100 mm; flow rate, 0.2 mL min^−1^; detection, UV 240 nm; temperature, 40 °C; mobile phase, tetrahydrofuran/MeOH = 1/1 (v/v).

The MIP using AcT4, 9G, and 4Vp as a template, a crosslinker, and a functional monomer, respectively, showed the greatest retention selectivity toward the template molecule. Furthermore, the molecular recognition ability could have shown like an induce-fit type recognition. Finally, to evaluate the retention selectivity to a variety of compounds, the 9G-MIP packed in column was analyzed by HPLC with several TR active and non-active compounds as analytes. In this evaluation, we selected the analytes based on our previous study, which showed TR active and non-active compounds by the yeast assay.^37,38^ The chemical structures of the analytes are summarized in Fig. S1† and classified the chemicals into TR non-active, TR weak-active, and TR strong-active. The results of retention factors of each analyte are summarized in Fig. 5. None of the analytes were retained on the 9G-NIP, and correlation between retention of each analyte and both chemical structures/the strength of TR activity was not observed. In the case of the 9G-MIP, the analytes having strong TR activity showed higher retention, and thus, slightly linear correlation between the strength of TR activity and retention of each analyte on the MIP was confirmed. Whereas, quite a few retentions were observed with TR weak-active compounds.

Aside from the results above, the result of AcT4 is not indicated in this figure. The retention factor of AcT4 on the MIP was much higher than any other analytes, the value was 18.2, although the TR activity was not stronger (24 nM, EC × 10) than other analytes. This result indicated that the 9G-MIP prepared with AcT4 has significantly strong recognition ability to the template molecule. However, the MIP constructed suitable molecular recognition sites for the TR strong-active compounds involving specific acidic groups at either end of the structure as well as AcT4. Thus, the MIP showed the selective retentions only toward the TR active compounds, suggesting that the MIP had molecular recognition similar to that of the receptor.

## Conclusion

In conclusion, we revealed the unique molecular recognition ability using the MIP with a flexible crosslinker. This study firstly found the presence of the suitable length of the spacer in a crosslinker during the MIP preparation for the accurate molecular recognition. Both the batch adsorption and HPLC analyses showed the presence of the highly selective recognition sites for the template molecule, AcT4. Furthermore, according to the thermodynamic approaches, the MIP also indicated the induced-fit type molecular recognition, briefly memorizing the stable molecular recognition sites at polymerization temperature. As no studies of the MIP have reported such results, therefore, our study will contribute to further investigations for highly accurate artificial receptors using MIPs.

## Author contributions

Experiments were designed by T. Kubo and D. Nakajima. Polymer preparation, characterization, and HPLC analyses were carried out by M. Yagishita and T. Tanigawa. The manuscript was written by T. Kubo and S. K.-Yamada.

## Conflicts of interest

There are no conflicts to declare.

## Supplementary Material

RA-014-D3RA08854E-s001
