# Supplementary material for: Enhanced molecular recognition with longer chain crosslinkers in molecularly imprinted polymers for an efficient separation of TR active substances
Source: RSC Adv. 2024 Apr 15;14(17):12021–9. doi: 10.1039/d3ra08854e (PMC11017824; doi:10.1039/d3ra08854e)
Supplement: RA-014-D3RA08854E-s001 [file RA-014-D3RA08854E-s001.pdf]

Electronic Supplementary Material (ESI) for RSC Advances.
This journal is © The Royal Society of Chemistry 2024

Supplementary information

Enhanced Molecular Recognition with Longer Chain Crosslinkers in Molecularly Imprinted Polymers for An Efficient Separation of TR Active Substances

Takuya Kubo,[*,a] Mayuko Yagishita,[b] Tetsuya Tanigawa,[a] Sayaka Konishi-Yamada [a] and Daisuke Nakajima,[c]

[a] *Department of Material Chemistry, Graduate School of Engineering, Kyoto University, Katsura, Nishikyo-ku, Kyoto 615-8510, Japan*
[b] *Department of Life and Environmental, Science, Prefectural University of Hiroshima, Shobara City, Hiroshima 727-0023, Japan*
[c] *Health and Environmental Risk Division, National Institute for Environmental Studies (NIES), Tsukuba City, Ibaraki 305-8506, Japan*

Corresponding author
Takuya KUBO
E-mail: kubo.takuya.6c@kyoto-u.ac.jp
orcid.org/0000-0002-9274-3295

Table of Contents

Chemicals and reagents
Instruments
Fig. S1. Chemical structures of crosslinkers, monomers, template, and analytes.
Table S1. Composition of the prepared MIPs and NIPs.
Fig. S2. Chromatograms with the MIP-packed columns.
Fig. S3. Chromatograms for TRIAC using the TRIAC-MIPs/NIPs packed columns.

Experimental

Chemicals

Dimethyl sulfoxide (DMSO), acetonitrile (MeCN, HPLC grade), methanol (MeOH, HPLC grade), acrylamide, formic acid, acetone, and distilled water were purchased from Nacalai Tesque, Inc. (Kyoto, Japan), 2,2'-azobis(2,4-dimethylvaleronitrile) (ADVN), ethylene glycol dimethacrylate (EDMA, distillation), bisphenol A (BPA), and hydrochloric acidwere from FUJIFILM Wako Pure Chemical Co. (Tokyo, Japan), 4-vinylpyridine (4Vp), triiodothyronine (T3), 17β-estradiol (E2), trimethylamine, tetrabromobisphenol A (TBBPA), *N*-succinimidyl acetate, 3, 5, 3'-triiodothyroacetic acid (TRIAC), and tetrachlorobisphenol A (TCBPA) were from Tokyo Chemical Industry Co., Ltd. (Tokyo, Japan), L-thyroxine (T4) and 3,5-dimethyl-4-(4'-hydroxy-3'-isopropylbenzyl)-phenoxy acetic acid were from Sigma-Aldrich (St. Louis, MO, USA), *N*-acetyl thyroxine (AcetylT4) was from Toronto Research Chemicals (North York, Canada), diiodothyropropionic acid (DITPA) was from Cayman Chemical Company (Ann Arbor, MI, USA). PEG dimethacrylate (1G, 4G, 9G, 14G, and 23G) was kindly donated from Shin-Nakamura Chemical (Wakayama, Japan) and utilized as received.

Instruments

Shimadzu HPLC systems (Kyoto, Japan) were employed, including an LC-30AD as a pump, a CTO-20AC as a column oven, and an SPD-M20A as a detector for protein analyses, and an LC-10Advp as a pump, a CTO-10Acvp as a column oven, and an SPD-M10Avp as a detector for purification of the synthesized compounds, an LC-30Adas a pump, a CTO-20AC as a column oven, an SPD-M20A as a photo diode array detector, an LCMS-8050 as a mass spectrometer. Morphological characterization was performed by a nitrogen gas adsorption analysis, Gemini VII 2390 (Micromeritics Instrument Co. Norcross, GA, USA).

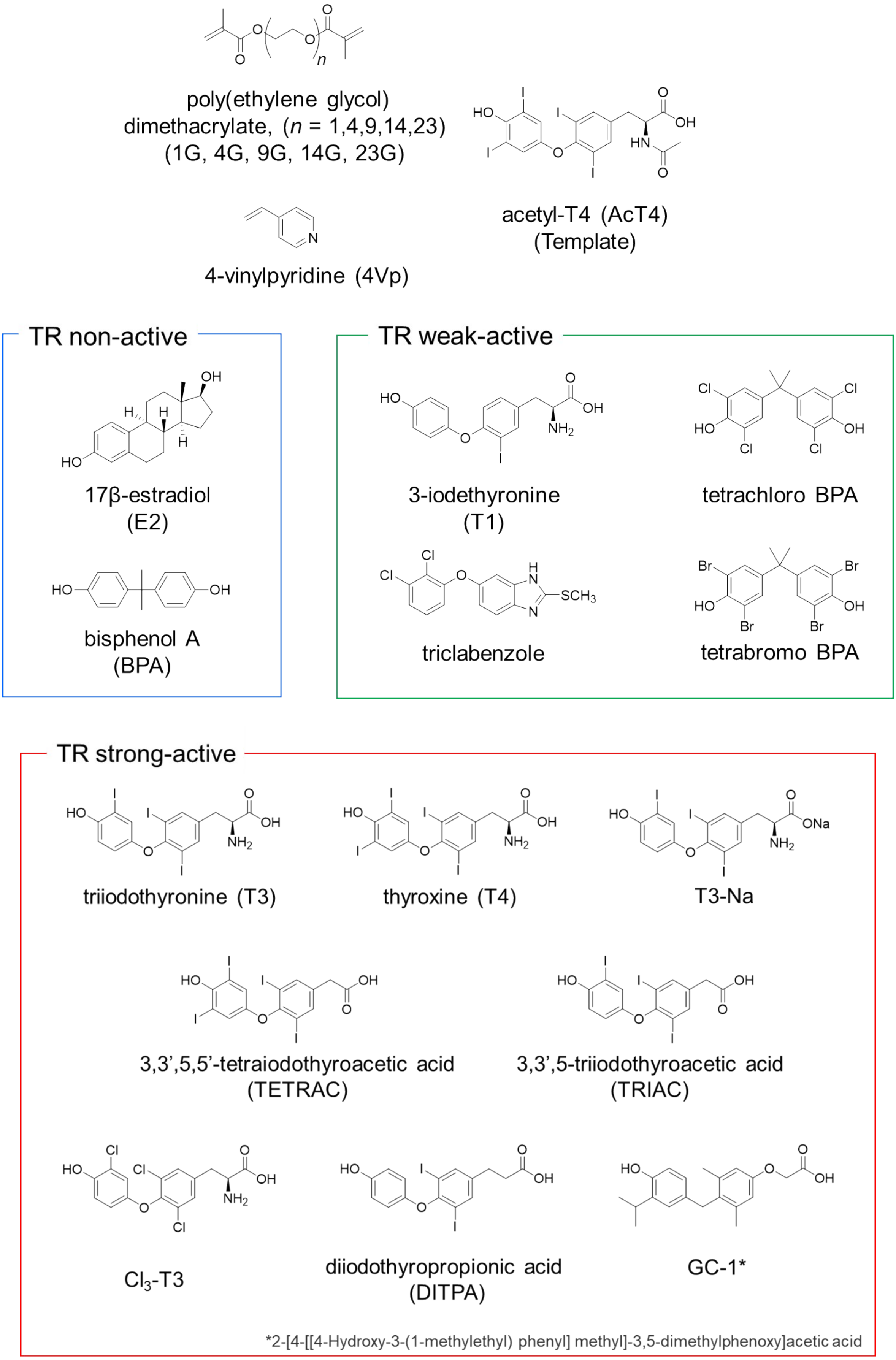

Fig. S1. Chemical structures of crosslinkers, monomers, template, and analytes.

Table S1. Composition of the prepared MIPs and NIPs.

Entry	EDMA Base gel	Crosslinker	Functional monomer	Template
1G-NIP	0.5 g	1G, 50 mg	4Vp, 112 μmol	------------
1G-MIP		1G, 50 mg		AcT4, 28 μmol
4G-NIP		4G, 50 mg		------------
4G-MIP		4G, 50 mg		AcT4, 28 μmol
9G-NIP		9G, 50 mg		------------
9G-MIP		9G, 50 mg		AcT4, 28 μmol
14G-NIP		14G, 50 mg		------------
14G-MIP		14G, 50 mg		AcT4, 28 μmol
23G-NIP		23G, 50 mg		------------
23G-MIP		23G, 50 mg		AcT4, 28 μmol
9G-MIP-less		9G, 10 mg		AcT4, 28 μmol
9G-MIP-more		9G, 100 mg		AcT4, 28 μmol
1G-MIP-TRIAC		1G, 50 mg		TRIAC, 28 μmol
4G-MIP-TRIAC		4G, 50 mg		TRIAC, 28 μmol
9G-MIP-TRIAC		9G, 50 mg		TRIAC, 28 μmol

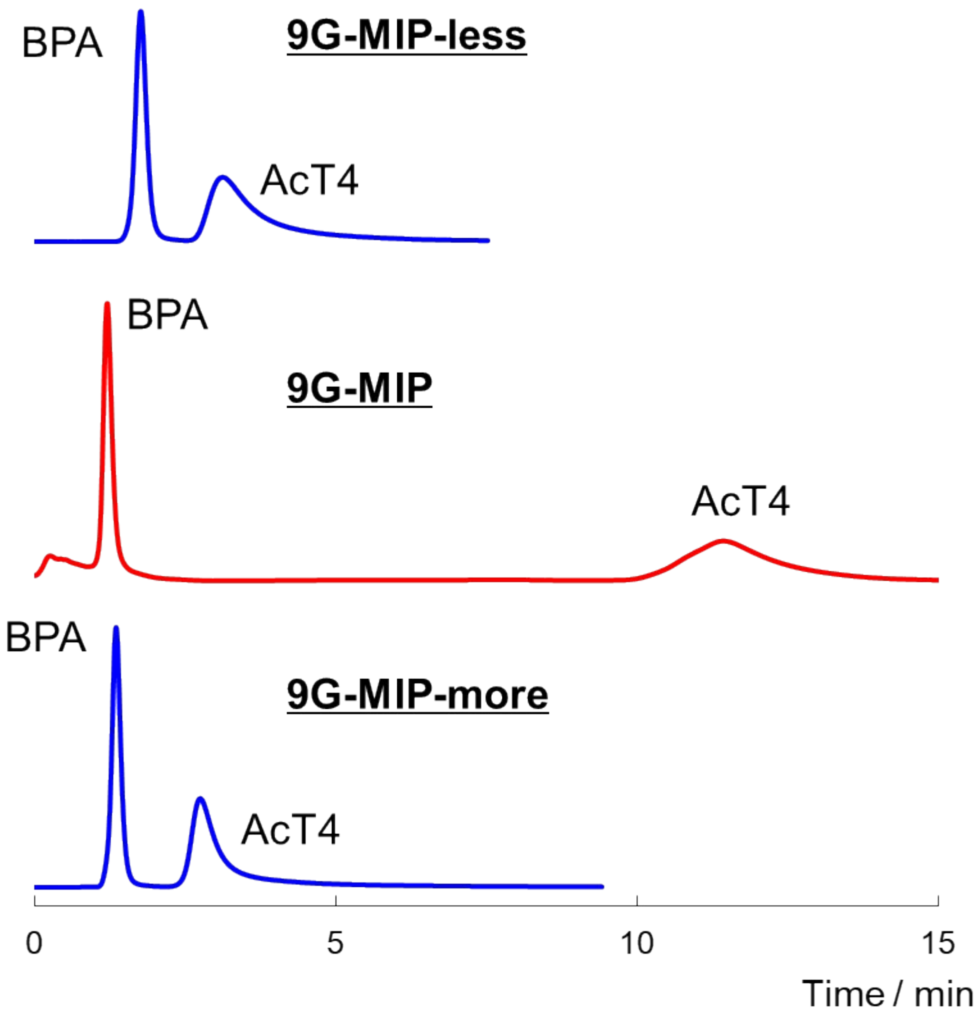

Fig. S2. Chromatograms with the MIP-packed columns.

HPLC conditions

Analytes, BPA + AcT4 (0.1 mg/mL, 2 µL); column size, 2.0 mm i.d. × 50 mm; flow rate, 0.2 mL/min; detection, UV 240 nm; temperature, 40 °C; mobile phase, 0.2% HCOOH in 90%MeOH / acetonitrile = 10 / 90 (v/v).

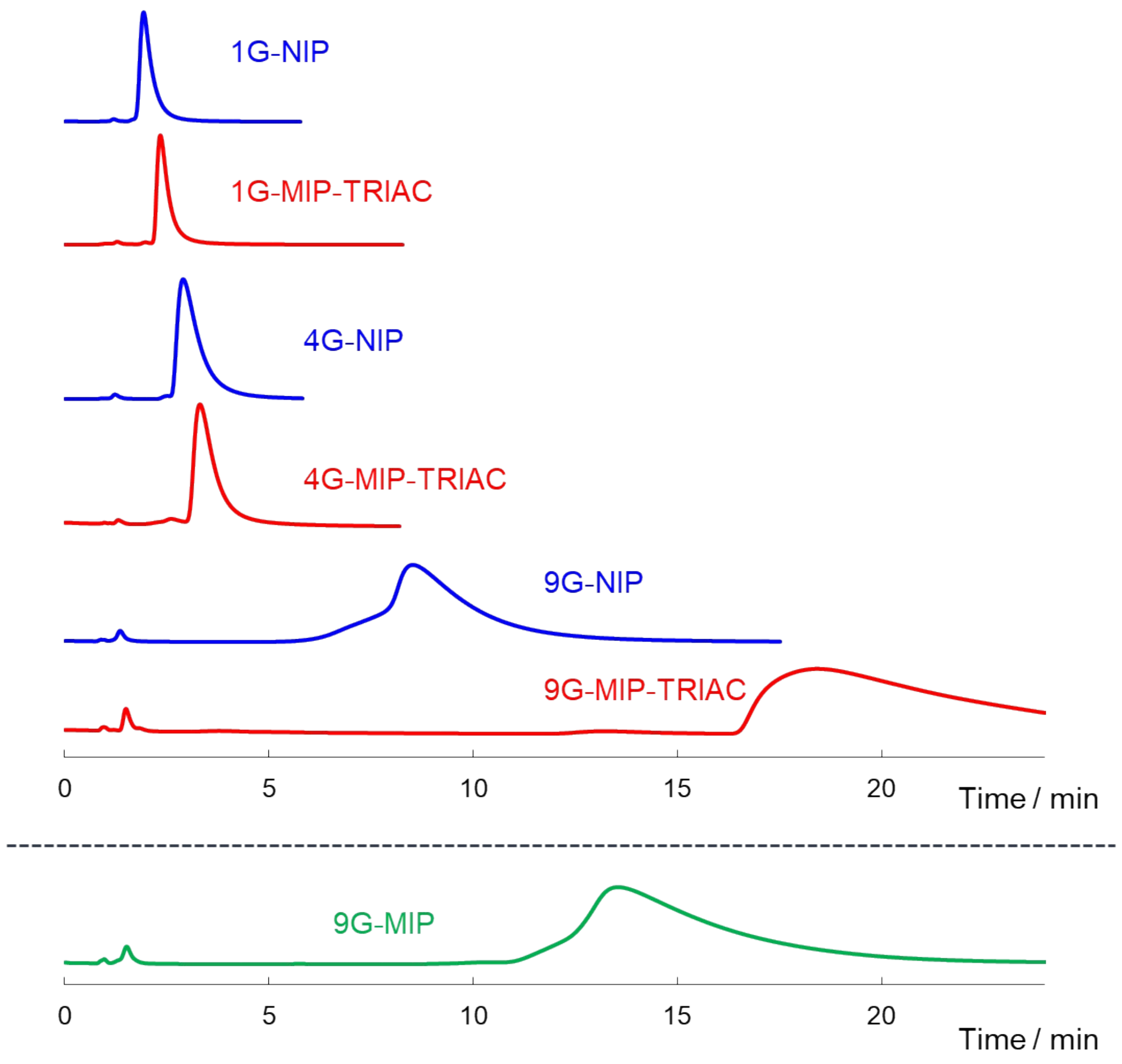

Fig. S3. Chromatograms for TRIAC using the TRIAC-MIPs/NIPs packed columns.

HPLC conditions

Analytes, TRIAC (0.1 mg/mL, 5 μL); column size, 2.0 mm i.d. × 50 mm; flow rate, 0.2 mL/min; detection, UV 240 nm; temperature, 40 °C; mobile phase, acetonitrile.
